# Feature matching based on local windows aggregation

**DOI:** 10.1016/j.isci.2024.110825

**Published:** 2024-08-28

**Authors:** Yuan Guo, Wenpeng Li, Ping Zhai, Lanlan Wu

**Affiliations:** 1 Heilongjiang University, No. 74 Xuefu Road, Harbin 150080, Heilongjiang, China; 2 Qiqihar University, No. 42 Wenhua Street, Qiqihar 161006, Heilongjiang, China; 3 Anhui Wenda University of Information Engineering, No. 3 Forest Avenue, Hefei 231201, Anhui, China

**Keywords:** Applied sciences, Computer science, Network modeling

## Abstract

The core goal of feature matching is to establish correspondences between two images. Current methods without detectors achieve impressive results but often focus on global features, neglecting regions with subtle textures and resulting in fewer matches in areas with weak textures. This paper proposes a feature-matching method based on local window aggregation, which balances global features and local texture variations for more accurate matches, especially in weak-texture regions. Our method first applies a local window aggregation module to minimize irrelevant interference using window attention, followed by global attention, generating coarse and fine-grained feature maps. These maps are processed by a matching module, initially obtaining coarse matches via the nearest neighbor principle. The coarse matches are then refined on fine-grained maps through local window refinement. Experimental results show our method surpasses state-of-the-art techniques in pose estimation, homography estimation, and visual localization under the same training conditions.

## Highlights

• We propose an optimization method to obtain more accurate sub-pixel matching positions
• We designed a local window aggregation module to obtain better image feature points
• Perform outstanding match in weak texture region integrating coarse and fine features

## Graphical abstract

**Figure:**
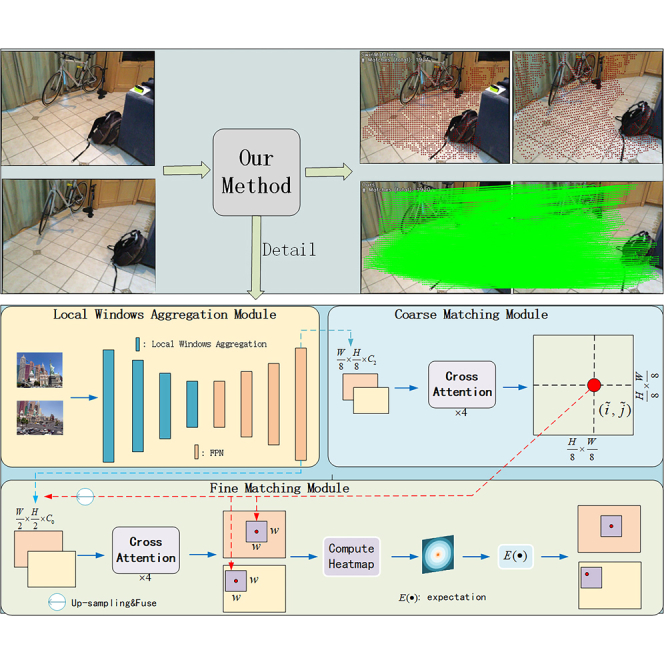

## Introduction

Feature matching is a fundamental task in computer vision, aiming to establish correspondences between features in pairs of images. It serves as a cornerstone for many three-dimensional vision tasks, including structure-from-motion (SfM),^1^ 3D reconstruction,^2^ visual localization,^3^^,^^4^ and pose estimation.^5^^,^^6^ Feature matching also finds interdisciplinary applications. In the medical field, image-matching techniques are used to register medical images taken by different devices or, at other times, allow doctors to analyze images from various perspectives and improve diagnostic accuracy comprehensively. For microscope images, feature matching is employed to detect and track changes in cells or subcellular structures, aiding research on cell behavior and biological processes. In environmental science, feature matching techniques can be used to compare glacier images taken at different times to analyze glacier advancement or retreat and study the impact of climate change on glaciers. It can also detect and track cracks and fissures in ice caps to predict potential iceberg calving events. In the preservation of cultural heritage, feature matching technology can match and align artifact fragments, assisting restorers in reconstructing damaged historical artifacts. Additionally, it can integrate different images of artifacts to create high-precision 3D digital models for preservation and display. Given the critical role of feature matching in these applications, it has garnered widespread research attention and has driven progress in numerous academic studies.^7^^,^^8^^,^^9^^,^^10^^,^^11^ However, feature matching faces innumerable challenges, including variations in lighting conditions, scale changes, poor texture, and repetitive patterns, all of which significantly increase the difficulty of obtaining consistent and accurate matching results.

**Figure 1:**
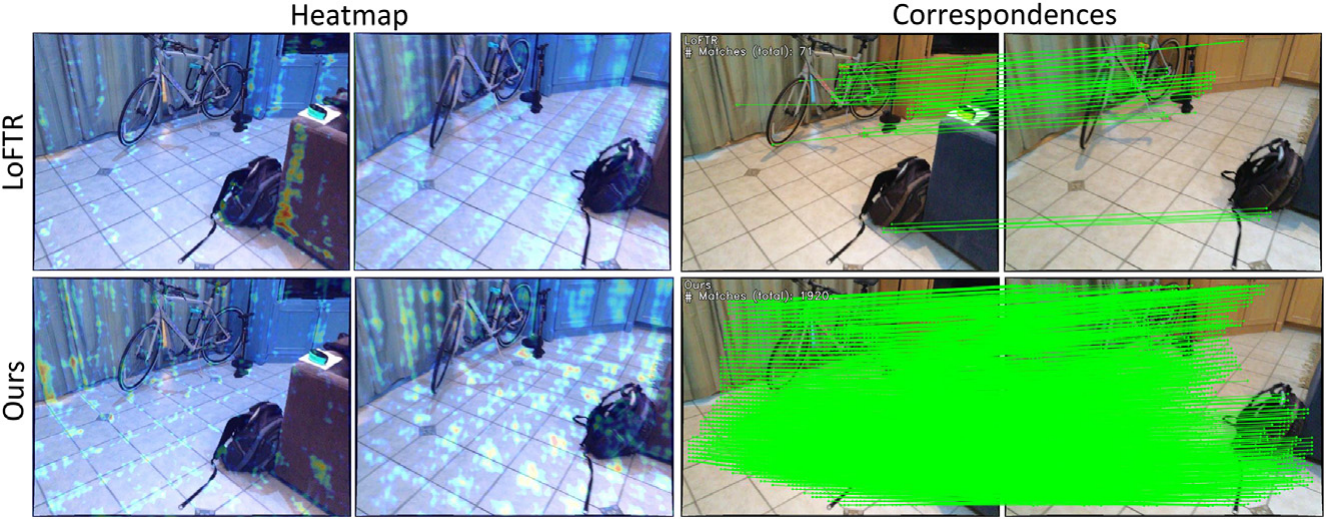
Our method can detect more correspondences compared to LoFTR

To address the multiple challenges in feature matching, scholars have proposed various methods, mainly detector-based matching methods^7^^,^^9^^,^^12^^,^^13^^,^^14^^,^^15^ and detector-free matching methods.^8^^,^^10^^,^^11^^,^^16^^,^^17^^,^^18^ Detector-based methods first utilize keypoint detectors to identify keypoints between images and then establish correspondences between these keypoints. The quality of keypoints significantly influences the matching performance, so many studies have focused on optimizing keypoint detection through multi-scale detection^19^ and reliability verification^9^ to improve matching performance while maintaining high computational and memory efficiency. However, these methods often struggle to find reliable matches in textureless regions where keypoints are challenging to detect. In contrast, detector-free matching methods do not require keypoints to be detected beforehand; instead, they directly attempt to establish pixel-level correspondences between features, enabling matching in textureless regions. In recent years, Transformer-based matching methods have gained widespread use due to their advantage in capturing long-range dependencies.^11^^,^^16^^,^^20^^,^^21^ Representative works such as LoFTR^11^ utilize a linear transformer^22^ in the coarse matching stage to obtain and refine global features. COTR^23^ iteratively computes shared visible regions through attention mechanisms to address the scale variation issue. These Transformer-based methods demonstrate the effectiveness of attention mechanisms in feature matching. However, recent research^24^^,^^25^ suggests that Transformers may lack spatial perception bias in continuous dense prediction tasks, which could lead to inconsistent matching results. Previous methods used global attention mechanisms, leading to ignoring local feature information and poor performance matching weak texture regions. Our method differs from earlier methods by focusing more on the local features of the image, as the nature of image features is significantly localized. At the same time, we do not want to lose global feature information, as feature matching requires guidance from global features. Therefore, we propose a local window-based feature matching method. This method first processes the image through a local window aggregation module consisting of global and local attention mechanisms. This allows us to obtain local features while retaining global feature information. The local window aggregation module generates coarse-grained and fine-grained feature maps. Since feature matching requires comparing and matching features from two images, these feature maps are further processed through a feature matching module composed of cross-attention mechanisms, and coarse-grained matching is performed using a mutual nearest neighbor principle. To obtain accurate sub-pixel matching information, we finally refined the coarse-grained matching results on the fine-grained feature map using cross-attention processing in local windows to get the final matching results. Figure 1 demonstrates the superiority of our method. The heatmap shows we capture features more effectively (darker colors represent better performance). Additionally, the matching results show that our method yields more correspondences.

The contributions of our method could be summarized into 4-fold.

(1) Proposed a feature matching method based on local windows, consisting of a local window aggregation module, a coarse-grained feature matching module, and a fine-grained feature matching module.
(2) We designed a module for local window aggregation that combines local windows with global attention intersection. This approach ensures that local consistency is maintained without being influenced by irrelevant regions while also considering global context, which enables the detection of more correspondences, especially in weak texture areas.
(3) We designed a matching module that progresses from coarse to fine. Initially, coarse matches are obtained through cross-attention applied to coarse-grained features. Subsequently, these coarse matches are fused with fine-grained features using cross-attention. Finally, refinement is performed on the fine-grained level using the coarse matching results to achieve the ultimate sub-pixel level matching accuracy.
(4) Extensive experimental results on challenging benchmarks indicate that our proposed method outperforms state-of-the-art image-matching approaches.

### Related work

Feature matching can be broadly categorized into two main types: detector-based methods and detector-free methods. Detector-based feature matching typically consists of three main stages: feature detection, description, and matching. Manually designed feature detectors such as SIFT^26^ and ORB^27^ are well-known in feature detection. In recent years, learning-based methods^7^^,^^9^^,^^12^^,^^13^^,^^26^^,^^27^^,^^28^ have demonstrated superior performance to traditional handcrafted methods. For instance, D2Net^13^ combines feature detection and description stages, while R2D2^9^ aims to train a network to identify reliable and repeatable features. Additionally, SuperGlue^15^ proposes an attention-based graph neural network (GNN) that optimizes extracted features through the alternating update of self-attention and cross-attention. However, detector-based methods rely on local feature extractors, which may limit performance in challenging scenarios such as repetitive textures, weak textures, and illumination variations. In contrast, detector-free methods do not rely on local feature detectors; instead, they directly find dense feature matches between pixels. This approach circumvents the limitations of traditional feature extraction, allowing for more flexible and extensive matching in various complex environments. Learning-based methods were first adopted in literature,^29^^,^^30^ where pixel-level feature descriptors were learned using contrastive loss. Similar to detector-based methods, matching of dense descriptors is typically achieved through nearest neighbor search. NCNet^10^ adopts a different strategy by directly learning dense correspondences through an end-to-end learning approach. This method constructs a 4D cost volume to enumerate all potential image matches and normalizes them through 4D convolution to ensure neighborhood consistency among matches. Sparse NCNet^18^ improves upon NCNet^10^ by introducing sparse convolution to enhance efficiency. Following this line of research, DRC-Net^8^ utilizes CNN feature maps of two different resolutions to construct two 4D matching tensors, which are then fused to achieve high-confidence feature matching. Additionally, a coarse-to-fine matching strategy is proposed to improve the accuracy of dense matching. While the 4D cost volume considers all possible matches, the receptive field of 4D convolution remains limited to the neighborhood region of each match. In recent years, Transformer models^31^ have garnered widespread attention in the computer vision domain. When handling visual tasks such as image classification,^32^^,^^33^^,^^34^ object detection,^35^^,^^36^^,^^37^ and image segmentation,^38^^,^^39^ Transformers leverage their global interaction capability to explore key regions in images. Due to their outstanding performance, Transformer techniques have also been applied in image feature matching.^11^^,^^15^^,^^21^ Despite significant achievements, the original attention mechanism of Transformers incurs high computational costs when processing high-resolution images. Therefore, various approximation methods^20^^,^^22^^,^^40^^,^^41^ have been proposed to reduce costs, often at the expense of performance. For example, linear attention mechanisms^22^ approximate the softmax function using the ELU^42^ function to reduce the computational complexity linearly, albeit weakening the model’s focusing ability. ASpanFormer^16^ introduces an adaptive attention breadth selection method, which, while flexible, often overlooks the importance of local consistency. Additionally, Transformers may sometimes ignore local feature information in image tasks.^43^ To overcome these limitations, we propose a novel strategy that avoids additional computational and memory overheads by aggregating features using local windows to maintain local consistency, effectively enhancing the accuracy and efficiency of feature matching.

### Method

**Figure 2:**
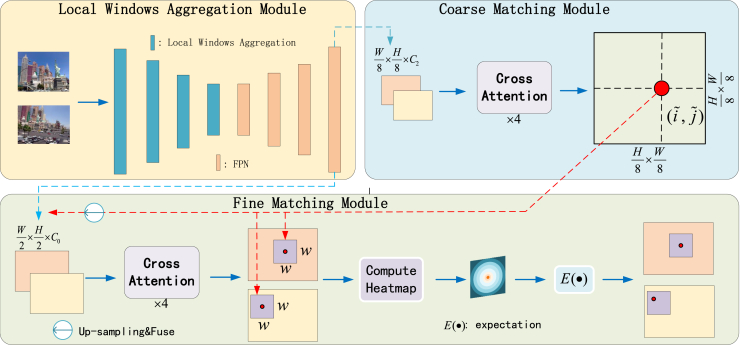
The overall process of our method

Our method follows the overall process outlined in Figure 2 to perform feature matching between two images. Our method mainly consists of three modules: the local windows aggregation module, the coarse matching module, and the fine matching module. Below, we briefly introduce the entire process. Given images $I^{A}$ and $I^{B}$, first, we extract multi-scale feature maps for each image using the local windows aggregation module. We denote the feature map of size 1/i as $F^{1/i}=\left\{F_{A}^{1/i},F_{B}^{1/i}\right\}$. Next, we input $F^{1/8}$ into the coarse matching module for coarse-grained feature matching. We use the nearest neighbor principle to obtain a confidence matrix $P_{c}$ and predict coarse-grained matches $M_{c}$ based on a confidence threshold. Finally, we input $F^{1/2}$, $F^{1/8}$ and the coarse-grained matches $M_{c}$ into the fine matching module. We upsample the $F^{1/8}$ and fuse them with the $F^{1/2}$ before performing fine-grained matching. We crop local windows from the $F^{1/2}$ and compute the spatial expectation coordinates of the two-dimensional heatmap for each local window to obtain the final matching results $M_{f}$.

#### Local windows aggregation

**Figure 3:**
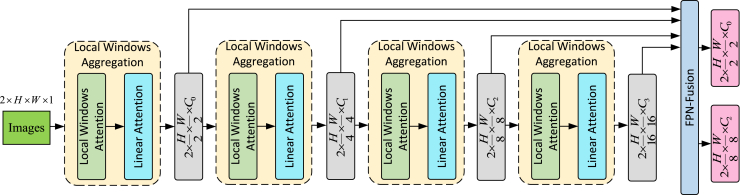
The structure of the local window aggregation module

The local windows aggregation (LWA) module, as shown in Figure 3, takes the input image $I\in R^{2\times H\times W\times 1}$ (2, H, W, 1, representing the number of images, height, width, and the number of channels) and undergoes four rounds of local window aggregation processing and feature pyramid network (FPN) processing to obtain $F^{1/2}$ and $F^{1/8}$. The $C_{0}$, $C_{1}$, $C_{2}$, and $C_{3}$ represent the dimensions of the feature maps. Initially, the image is a grayscale image with only one dimension. After the first local window aggregation, the feature dimension changes from 1 to $C_{0}$, halving the height and width. After the second local window aggregation, the feature dimension changes from $C_{0}$ to $C_{1}$, and the height and width are halved again. After the third local window aggregation, the feature dimension changes from $C_{1}$ to $C_{2}$, and the height and width are halved again. After the fourth local window aggregation, the feature dimension changes from $C_{2}$ to $C_{3}$, and the height and width are halved once more. Finally, the four feature maps are fused through a feature pyramid, outputting two feature maps for subsequent coarse-grained and fine-grained feature matching. The dimensions of these two feature maps are $C_{0}$ and $C_{2}$, respectively. We denote the processing at each stage as $LWA_{i}\left(\cdot \right)$ and the FPN as FPN(·). The local window aggregation processing is represented as:

(Equation 1) $$I_{i}=LWA_{i}\left(I_{i}\right),i=1,2,3,4$$

(Equation 2) $$\left\{\left(F_{A}^{1/8},F_{B}^{1/8}\right),\left(F_{A}^{1/2},F_{B}^{1/2}\right)\right\}=FPN\left(I_{i}\right),i=1,2,3,4$$

Finally, we obtain feature maps of original image sizes 1/8 and 1/2.

In local window aggregation, the attention mechanism plays a central role. Ordinary attention calculation requires three inputs: Q (query), K (key), and V (value). The attention output is a weighted sum, where the weight matrix is determined by Q and its corresponding K. This process can be described as:

(Equation 3) $$Attention\left(Q,K,V\right)=SoftMax\left(QK^{T}\right)V$$

However, in visual tasks, the size of the weight matrix $SoftMax\left(QK^{T}\right)V$ grows quadratically with the increase in image resolution. When the image resolution is high, ordinary attention’s memory and computational costs become prohibitive. To address this issue, linear attention has been proposed,^22^ which replaces the softmax operation with the product of two kernel functions:

(Equation 4) $$Linear\_attention\left(Q,K,V\right)=\phi \left(Q\right)\left(\phi \left(K^{T}\right)V\right)$$

Here, ϕ(·)=elu(·)+1. Since the number of feature channels is much smaller than the number of pixels, the computational complexity decreases from quadratic to linear. Therefore, we adopt linear attention for global attention.

**Figure 4:**
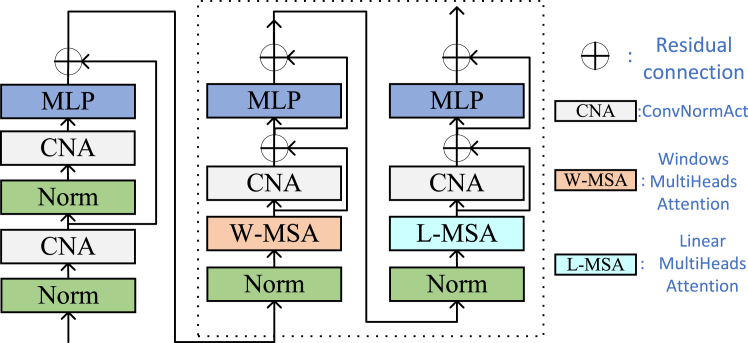
The specific implementation details of the local window aggregation

The local attention mechanism is inspired by the Swin Transformer,^40^ but unlike it, we do not use the shifting window operation and instead replace it with global attention. Additionally, we do not require the image height and width to be exact multiples of the window size; if they are not divisible, we pad the image accordingly. For an image input of size H×W×C, we first reshape it into a feature map of size $\frac{HW}{M^{2}}\times M^{2}\times C$ and divide this feature map into M×M non-overlapping local windows, where $\frac{HW}{M^{2}}$ represents the number of windows. Then, self-attention is computed separately for each window. The matrix calculation method for local window features $X\in R^{M^{2}\times C}$ is specific and used to implement local attention:

(Equation 5) $$Q=XP_{Q},K=XP_{K},V=XP_{V}$$

where $P_{Q},P_{K},P_{V}$ are projection matrices shared across different windows. Typically, $Q,K,V\in R^{M^{2}\times d}$. The attention matrix calculated through the self-attention mechanism within the local window is:

(Equation 6) $$Attention\left(Q,K,V\right)=SoftMax\left(QK^{T}/\sqrt{d}+B\right)V$$

where d represents the number of heads in multi-head attention. We denote this as W-MSA, which stands for window multi-head attention mechanism. The specific steps are illustrated in Figure 4, starting with a convolution layer that increases the tensor channels while halving the dimensions. Subsequent processing involves local window attention and global attention, a step that can be repeated N times to enhance the feature extraction effect.

#### Coarse and fine matching module

Through the local window aggregation module, we obtain $F^{1/2}$ and $F^{1/8}$. Firstly, we perform cross-attention processing on the $F^{1/8}$ of the image pair, which enhances the effectiveness of coarse-grained feature matching. Then, on the processed $F^{1/8}$, we calculate the score matrix *S* between transformed features using $S\left(i,j\right)=\frac{1}{\tau }\cdot \left\langle {\tilde{F}}^{A}\left(i\right),{\tilde{F}}^{B}\left(j\right)\right\rangle$, where τ is a temperature coefficient. Subsequently, softmax is applied along both dimensions of *S* (referred to as double softmax) to obtain the probability of soft mutual nearest neighbor matching. When using double softmax, the matching probability $P_{c}$ is given by:

(Equation 7) $$P_{c}\left(i,j\right)=soft\;\max\;{\left(S\left(i,\cdot \right)\right)}_{j}\cdot soft\;\max\;{\left(S\left(\cdot ,j\right)\right)}_{i}$$

Based on the confidence matrix $P_{c}$, matches with confidence higher than the threshold $\theta_{c}$ are selected, and the Mutual Nearest Neighbor (MNN) criterion is further applied to filter potential outlier coarse matches. We represent the coarse match prediction as:

(Equation 8) $$M_{c}=\left\{\left(\tilde{i},\tilde{j}\right)|\forall \left(\tilde{i},\tilde{j}\right)\in MNN\left(P_{c}\right),P_{c}\left(\tilde{i},\tilde{j}\right)\geq \theta_{c}\right\}$$

After establishing coarse-grained matching, we merge the upsampled $F^{1/8}$, processed with cross-attention, with $F^{1/2}$ to obtain a new $F^{1/2}$. Then, we perform another round of cross-attention processing on the new $F^{1/2}$ to further enhance the effect of coarse-grained feature matching. We utilize an association-based approach to refine the coarse-grained matching to the original image resolution. For each coarse-grained match $\left(\tilde{i},\tilde{j}\right)$, we first locate its position $\left(\hat{i},\hat{j}\right)$ on $F^{1/2}$. Then, we crop out two sets of local windows of size w×w. Generating two transformed local feature maps, $F_{A}^{1/2}\left(\hat{i}\right)$ and $F_{B}^{1/2}\left(\hat{j}\right)$, with $\hat{i}$ and $\hat{j}$ as centers, we associate the central vector of $F_{A}^{1/2}\left(\hat{i}\right)$ with all vectors of $F_{B}^{1/2}\left(\hat{j}\right)$ to obtain a heatmap representing the probability of each pixel in the neighborhood of $\hat{j}$ matching with $\hat{i}$. By calculating the expectation on the probability distribution, we get the final position ${\hat{j}}^{\prime }$ on $I^{B}$ with sub-pixel accuracy. Summing up all matches $\left\{\left(\hat{i},{\hat{j}}^{\prime }\right)\right\}$ results in the final fine-grained matching, $M_{f}$.

#### Loss function

The final loss consists of coarse-grained loss and fine-grained loss: $L=L_{c}+L_{f}$. We have tested different loss weights, such as $L=L_{c}+2L_{f}$, $L=2L_{c}+L_{f}$ and $L=0.5L_{c}+L_{f}$. But the final training performance was slightly weaker than $L=L_{c}+L_{f}$. Therefore, in our final model, the weights for coarse-grained and fine-grained loss are equal.

The coarse-grained loss function is defined by the negative log likelihood loss returned from the double softmax operation on the confidence matrix $P_{c}$. The true label values for the confidence matrix during training are calculated using camera poses and depth maps. We define the true coarse-grained matches $M_{c}^{gt}$ as the mutual nearest neighbors of the two sets of 1/8 resolution networks. The distance between two networks is measured by their reprojection distance from their center positions. We minimize the negative log likelihood loss of the networks in $M_{c}^{gt}$.

(Equation 9) $$L_{c}=-\frac{1}{\left|M_{c}^{gt}\right|}\sum_{\left(\tilde{i},\tilde{j}\right)\in M_{c}^{gt}}\log\;P_{c}\left(\tilde{i},\tilde{j}\right)$$

For fine-grained refinement at the pixel level, we use the L2 loss. For each point $\hat{i}$ to be matched, we measure its uncertainty by computing the total variance of the corresponding heatmap, denoted as $\sigma^{2}\left(\hat{i}\right)$. The goal is to optimize the positions of fine-grained matches with lower uncertainty, resulting in the weighted loss function:

(Equation 10) $$L_{f}=\frac{1}{\left|M_{f}\right|}\sum_{\left(\hat{i},{\hat{j}}^{\prime }\right)\in M_{f}}\frac{1}{\sigma^{2}\left(\hat{i}\right)}∥{\hat{j}}^{\prime }-{\hat{j}}_{gt}^{\prime }{∥}_{2}$$

Here, ${\hat{j}}_{gt}^{\prime }$ is computed by mapping each $\hat{i}$ from $F_{A}^{1/2}\left(\hat{i}\right)$ to $F_{B}^{1/2}\left(\hat{j}\right)$ using the true camera poses and depth information. When calculating $L_{f}$, if the mapped position of $\hat{i}$ falls outside the local window of $F_{B}^{1/2}\left(\hat{j}\right)$, we ignore this pair $\left(\hat{i},{\hat{j}}^{\prime }\right)$. During training, gradients do not propagate back through $\sigma^{2}\left(\hat{i}\right)$.

### Experiments

We used the ScanNet^44^ dataset for indoor training and the MegaDepth^45^ for outdoor training. Drawing inspiration from the methods of LoFTR^11^ and SuperGlue,^15^ we trained our model by sampling image pairs with overlap scores ranging from 0.4 to 0.8. Our model utilized the AdamW optimizer with an initial learning rate of $3.5\times 10^{-4}$ and a batch size 1. The training was conducted over 35 epochs on 8 RTX 2080 Ti GPUs until convergence. The entire model underwent end-to-end training with randomly initialized weights. During training, the local window aggregation module was iterated four times with depths of (3, 3, 6, 9) respectively. Coarse-grained and fine-grained cross-attention was applied four times. We also set the parameter $\theta_{c}$ to 0.2, and the window sizes for the local aggregation module and fine-grained matching were set to 5×5.

#### Pose estimation

Camera pose estimation has a wide range of applications in various fields. In augmented reality, precise analysis of the camera’s position and orientation in space allows virtual objects to be accurately superimposed onto real-world scenes, enhancing the user’s perceptual experience. In minimally invasive surgery, camera pose estimation is used to track the position and orientation of surgical instruments in real time, assisting doctors in performing precise operations and improving the safety and efficacy of surgeries. Intelligent security systems it is employed for the automatic calibration and adjustment of surveillance cameras, thereby enhancing the level of intelligent management in public safety. Our method can achieve more accurate estimations in camera pose estimation, which in turn can help downstream applications (such as those mentioned previously) to operate more quickly and accurately.

**Figure 5:**
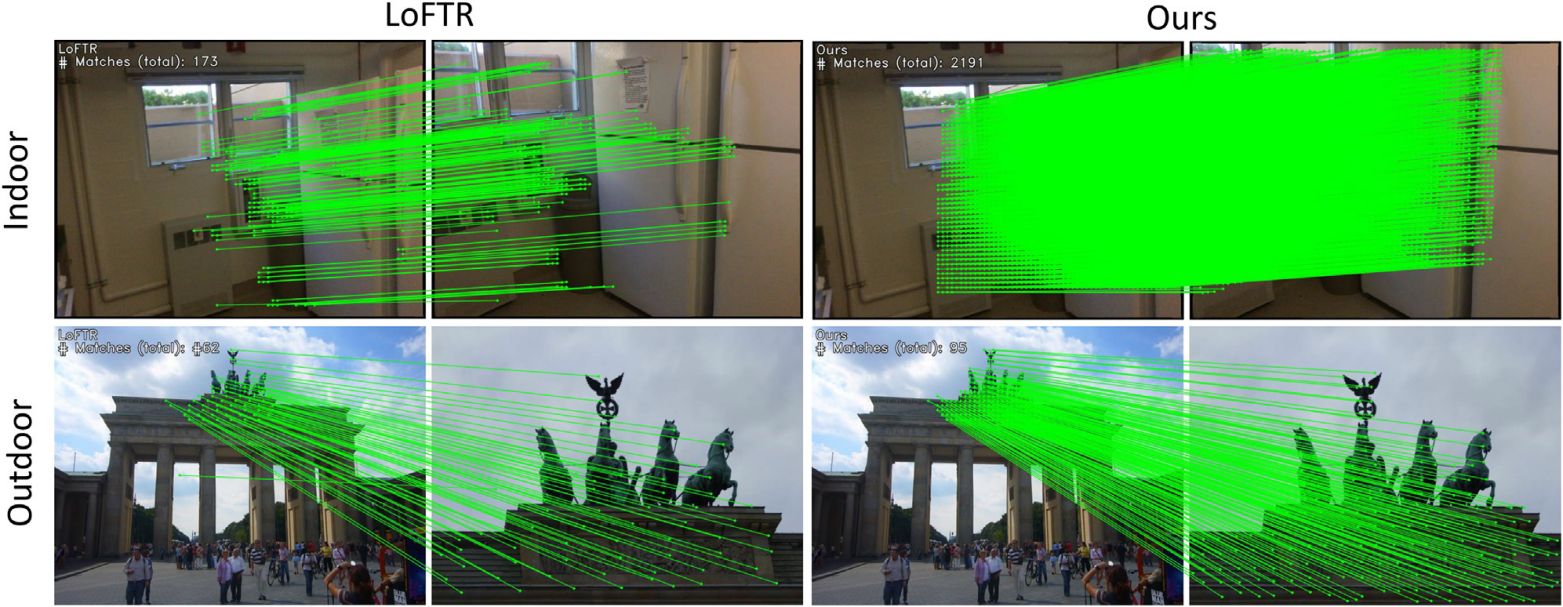
The feature matching performance of indoor and outdoor objects, our method can detect more correspondences

Indoor image matching faces challenges due to lack of texture, high self-similarity, and complex three-dimensional geometric structures. To demonstrate the effectiveness of our method in pose estimation for indoor scenes, we selected the ScanNet^44^ dataset and our dataset of weakly textured indoor wall images for experimentation. Table 1 compiles the area under the curve (AUC) of pose error and precision for various methods, where our method outperforms the previous best method, showing significant improvements in AUC@20° and precision, with a 1.14% increase in AUC@20° in particular. Experiments were conducted for outdoor pose estimation using the MegaDepth^45^ test dataset and our weakly textured outdoor wall images dataset. Table 2 similarly compiles the AUC of pose error and accuracy for various methods, with our method showing improvements in AUC@5°, AUC@10°, and precision, notably achieving a 1.8% increase in precision. Figure 5 intuitively shows the feature-matching performance of our method compared to others in indoor and outdoor scenarios, with our method achieving a higher number of correspondences.

**Table 1:** Indoor pose estimation on ScanNet

Category	Method	Pose estimation AUC			P
		@5°	@10°	@20°	
Detector-based	SIFT^26^+SuperGlue^15^	6.71	15.70	28.67	74.20
	SIFT^26^+IMP^46^	15.30	30.80	46.60	70.60
	Sp^7^+SuperGlue^15^	16.96	35.51	51.84	80.10
	SP^7^+IMP^46^	19.83	37.98	54.17	83.21
Detector-free	LoFTR^11^	21.95	40.51	57.96	88.09
	ASGT^47^	19.40	37.60	54.40	–
	DKM^48^	22.42	42.25	60.13	89.20
	Aspanformer^16^	22.59	42.39	61.20	88.73
	OAMatcher^49^	26.06	45.34	62.08	–
	Ours	22.57	42.95	62.79	89.95

**Table 2:** Outdoor pose estimation on MegaDepth

Category	Method	Pose estimation AUC			P
		@5°	@10°	@20°	
Detector-based	SIFT^26^+SuperGlue^15^	35.32	56.12	73.68	79.85
	SIFT^26^+IMP^46^	36.80	56.30	72.80	79.70
	SP^7^+SuperGlue^15^	42.18	61.16	75.96	85.19
	Sp^7^+IMP^46^	45.41	65.35	78.07	87.92
Detector-free	LoFTR^11^	49.80	66.20	79.20	93.39
	MatchFormer^21^	46.74	63.83	76.81	92.19
	DKM^48^	55.41	69.95	80.19	93.97
	Aspanformer^16^	53.32	69.58	81.21	94.28
	MR-Matcher^50^	55.96	72.15	83.45	–
	Ours	56.21	72.38	82.16	95.96

#### Homography estimation

Homography detection has significant applications in image processing and computer vision, especially in image registration and perspective transformation. In augmented reality, homography detection can be used to accurately overlay virtual objects onto real-world images, achieving realistic augmented reality effects. In robotic navigation, homography detection enables robots to plan paths and avoid obstacles in complex environments, thereby improving the accuracy and safety of navigation. In medical image processing, homography detection is employed to register images from different perspectives, providing more comprehensive diagnostic information. Our method achieves higher accuracy in homography estimation, which can enhance the outcomes of downstream tasks, offering richer information and more substantial presentation effects.

**Figure 6:**
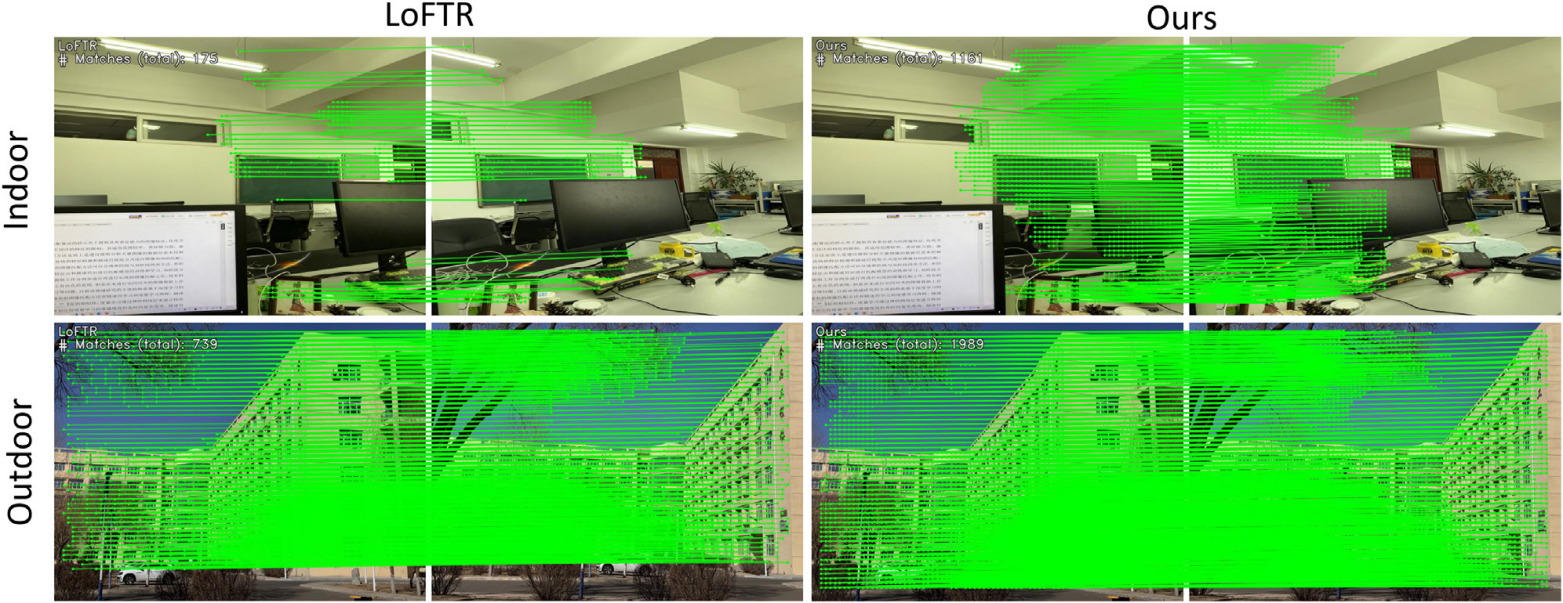
The feature matching performance on indoor and outdoor walls, our method can detect more weak texture correspondences

We conducted unified homography estimation tests on the HPatches^51^ dataset and our weakly textured indoor wall images dataset. In the tests, a reference image was paired with five other photos. Feature matching was performed for each image pair, and homography estimation was calculated using OpenCV, adopting the RANSAC method to enhance robustness. Table 3 compiles AUC of angular error, precision, and recall rate at different thresholds (3 pixels, 5 pixels, 10 pixels) for various methods. Our method outperformed others in AUC@3px, AUC@10px, precision, and recall rate, with the most remarkable improvement observed in AUC@10px, increasing by 2.5%. Figure 6 intuitively shows the matching performance of our method compared to others on indoor and outdoor weakly textured walls, with our method achieving a higher number of correspondences and detecting more weak texture information, such as performing feature matching on trees outdoors.

**Table 3:** Homography estimation on the HPatches and weakly textured indoor wall datasets

Category	Method	Homograph est. AUC			P	R
		@3px	@5px	@10px		
Detector-based	SIFT^26^+SuperGlue^15^	27.35	42.21	49.49	76.85	81.65
	SIFT^26^+IMP^46^	37.12	51.37	58.41	79.48	85.91
	SP^7^+SuperGlue^15^	42.75	55.18	60.89	82.18	90.75
	Sp^7^+IMP^46^	50.91	65.53	73.09	85.41	93.63
Detector-free	LoFTR^11^	60.52	70.94	79.76	89.17	97.29
	MatchFormer^21^	57.51	68.52	77.23	88.22	96.17
	DKM^48^	59.32	69.97	78.99	90.17	97.92
	Aspanformer^16^	60.16	69.63	80.21	89.58	97.73
	Ours	60.49	71.98	82.21	91.79	98.83

#### Visual localization

Visual localization plays a critical role in several fields. In autonomous driving, visual localization helps vehicles accurately determine their position on the road, enhancing driving safety and reliability. In robotic navigation, visual localization provides precise positional data in unknown environments, aiding robots in autonomous navigation. In virtual reality applications, visual localization helps systems accurately track the user’s position and posture, providing an immersive experience. By using our method, downstream tasks can achieve more precise localization, thereby improving the safety and reliability of applications.

We conducted experiments on visual localization. We evaluated our method on the long-term visual localization benchmark,^52^ which focuses on benchmarking visual localization methods under different conditions, such as day-to-night changes, scene geometry changes, and indoor scenes with large areas of texture-less regions. The evaluation was performed using the HLoc^53^ method on the InLoc^4^ dataset. Table 4 compiles the localization accuracy at angles of 10° and distances of 0.25m, 0.50m, and 1m under DUC1 and DUC2 conditions. Our method showed significant improvements, especially under DUC2, where the accuracy increased by an average of 1.65%.

**Table 4:** Visual localization using the HLoc method

Method	DUC1	DUC2
	(0.25m,10°)/(0.5m,10°)/(1.0m,10°)	
LoFTR^11^	45.4/70.1/81.8	52.1/72.5/83.7
ASGT^47^	53.0/73.7/86.4	52.7/76.3/84.0
DKM^48^	49.9/73.4/85.1	61.3/80.5/85.6
Aspanformer^16^	50.3/73.6/85.5	55.0/74.0/81.7
MR-Matcher^50^	50.2/75.8/85.9	56.5/75.6/86.3
OAMatcher^49^	51.0/75.8/84.8	55.0/77.1/84.7
Ours	53.0/73.5/87.1	62.7/81.9/87.1

#### Robustness experiments

**Figure 7:**
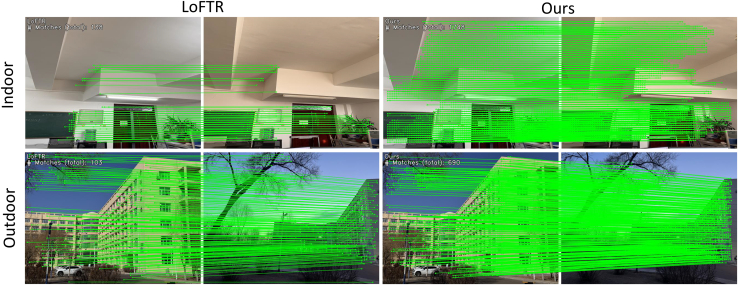
The feature matching performance on indoor and outdoor walls under different lighting conditions; our method can detect more correspondences

**Figure 8:**
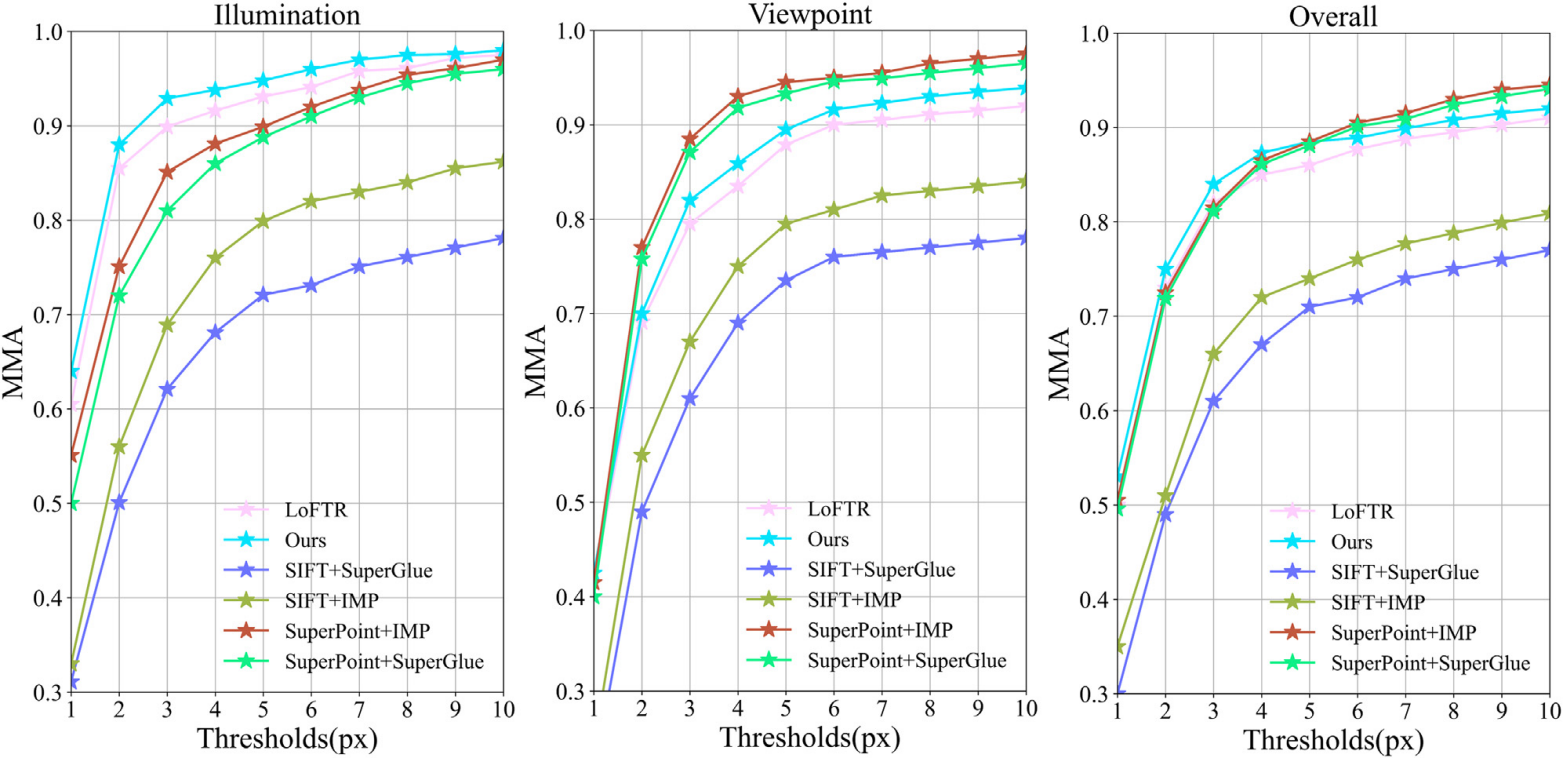
The average matching accuracy under illumination and viewpoint changes, as well as overall, on the HPatches and weakly textured indoor wall dataset

To evaluate the robustness of our method under changes in illumination and viewpoint, we conducted feature-matching tests using the HPatches^51^ and our weakly textured indoor wall dataset. We calculated the mean matching accuracy (MMA) and the correspondences from a 1-pixel to 10-pixel threshold.^21^ Table 5 and Table 6 compile the number of correspondences for different methods on the HPatches^51^ and our weakly textured indoor walls dataset, where our method performed the best, having the highest number of correspondences, averaging an improvement of 25.53% over the best. Figure 7 intuitively shows the matching performance of our method compared to others on indoor and outdoor weakly textured walls under different lighting conditions, with our method detecting a higher number of correspondences and more weak texture correspondences. Figure 8 compiles the average matching accuracy of different methods under various lighting conditions and viewpoint changes on the HPatches^51^ and our weakly textured indoor walls dataset. Under changes in illumination, our method had the best matching accuracy from 1 pixel to 10 pixels. In the case of viewpoint changes, our method had higher matching accuracy than other end-to-end methods, slightly lower than the detector-based method SuperPoint+IMP. Considering different lighting and viewpoint changes, below a 5-pixel threshold, our method exhibited the highest matching accuracy, and in the 6 to 10-pixel range, it was higher than other end-to-end methods and slightly lower than the detector method SuperPoint+IMP. These experimental results fully demonstrate the high robustness of our method.

**Table 5:** The number of correspondences on the HPatches indoor dataset

Category	Method	Matches
Detector-based	SIFT^26^+SuperGlue^15^	0.6K
	SIFT^26^+IMP^46^	0.4K
	SP^39^+SuperGlue^15^	1.1K
	Sp^7^+IMP^46^	1.3K
Detector-free	LoFTR^11^	1.5K
	MatchFormer^21^	1.9K
	Ours	2.2K

**Table 6:** The correspondences on the weakly textured indoor wall image dataset

Category	Method	Matches
Detector-based	SIFT^26^+SuperGlue^15^	0.3K
	SIFT^26^+IMP^46^	0.5K
	SP^7^+SuperGlue^15^	0.8K
	Sp^7^+IMP^46^	1.0K
Detector-free	LoFTR^11^	1.3K
	MatchFormer^21^	1.7K
	Ours	2.1K

#### Weak texture region matching

**Figure 9:**
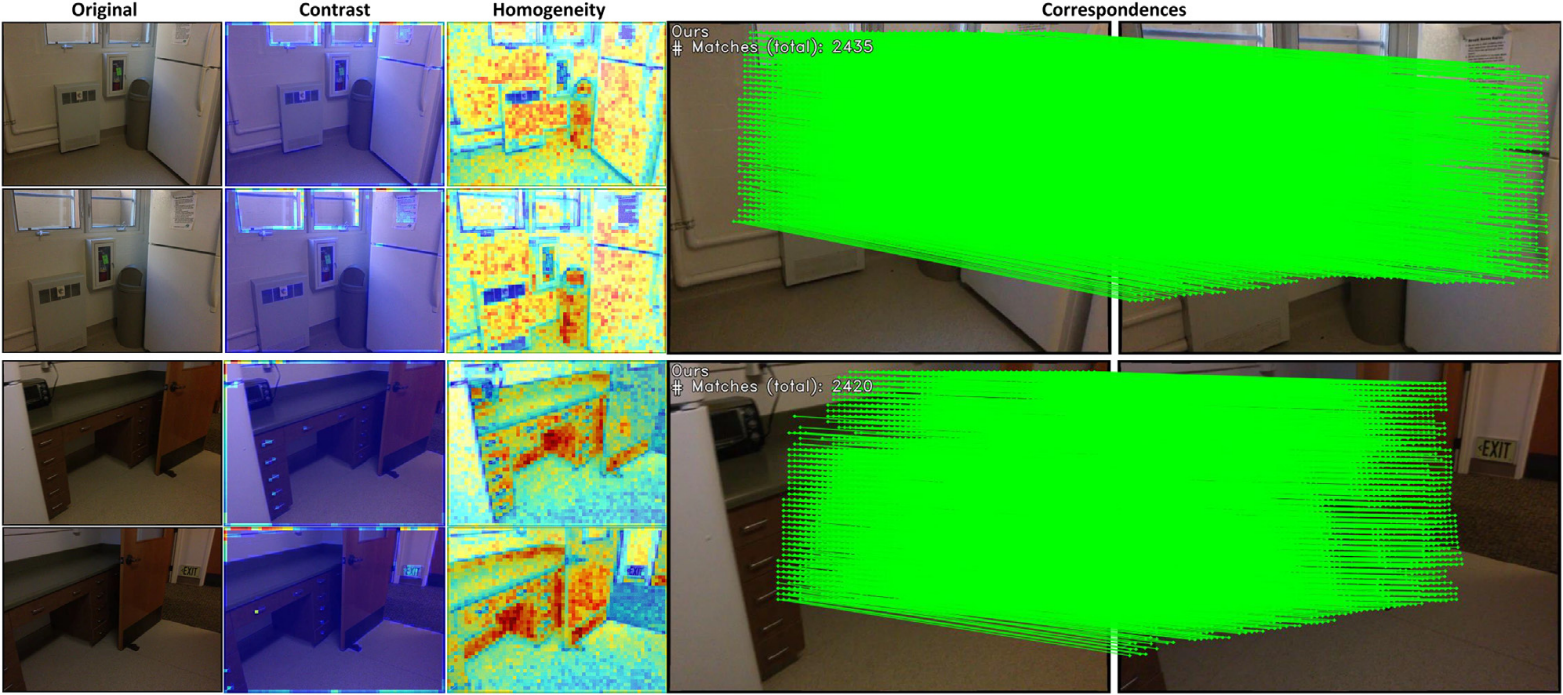
Feature matching in high and low texture regions

We validated the effectiveness of our method for matching weak texture regions on the ScanNet^44^ weak texture dataset. First, we captured the local texture features of the image using the Gray-Level Co-occurrence Matrix (GLCM). Then, we distinguished between high and low-texture regions by analyzing contrast and homogeneity. The comparison results are shown in Figure 9. In the contrast map, low-texture areas are represented in cool colors, while high-texture regions are shown in warm colors. Conversely, in the homogeneity map, low-texture areas are depicted in warm colors and high-texture regions in cool colors. Figure 9 demonstrates that most of the image consists of low-texture regions. Yet, our method effectively performs feature matching in these low-texture areas, with many matching feature points. Therefore, our method proves to be effective for matching between weak texture images.

#### Running time

The original Transformer has a high computational cost, so even though our method uses a modified version of the Transformer to accelerate computation, it still requires slightly higher computational resources. We calculated the number of parameters and GFLOPs for LoFTR,^11^ DKM,^48^ Matchformer,^21^ MR-Matcher,^50^ OAMatcher,^49^ and ours, as shown in Table 7. From this, we can see that we are not the best, but we are not the worst either. Overall, it is acceptable. We are also researching ways to reduce the computational load of the Transformer.

**Table 7:** Runtime comparison

Method	LoFTR^11^	MatchFormer^21^	DKM^48^	MR-Matcher^50^	OAMatcher^49^	Ours
Parameters	11.56M	27.11M	46.70M	11.92M	15.29M	12.94M
GLFOPs	349.54	90.93	336.80	349.10	384.74	206.15

#### Ablation study

The experiments in this subsection were all tested on the Scannet^44^ dataset. To verify the rationality of the model design, we conducted ablation experiments. We compared the impact of different local window sizes on the model, and the results are shown in Table 8. When the local window size increases, although the accuracy improves, the improvement is limited, and the number of matched feature points decreases sharply. Therefore, to obtain more matched feature points, we ultimately set the local window size to 5×5. Meanwhile, we also compared the impact of different modules on the model. W-MSA represents local window attention, L-MSA represents global attention, coarse-cross represents cross-attention processing in the coarse-grained matching stage, and fine-cross represents cross-attention processing in the fine-grained matching stage, with the number in parentheses indicating the number of cross-attentions. As shown in Table 9, we tested the effects of different module combinations, and the results without cross-attention processing in the matching stage were the worst. We also compared the number of cross-attentions in the matching stage. Simply increasing the number of cross-attentions does not improve the results and instead adds unnecessary parameters.

**Table 8:** The impact of Different Local Window Sizes on the Model

size of window	Pose estimation AUC@20°	Matches
5×5	62.79	2.318K
10×10	62.76	2.143K
20×20	62.80	1.994K
50×50	62.84	1.798K

**Table 9:** The impact of different modules on the model

W-MSA	L-MSA	Corase-cross	Fine-cross	Pose estimation AUC@20°	Precision
×	✓	✓	✓	61.69	88.12
✓	×	✓	✓	61.73	88.25
✓	✓	×	✓	58.54	82.93
✓	✓	✓	×	59.39	81.37
✓	✓	×	×	48.26	75.08
✓	✓	✓	✓	62.79	89.95

#### The differences between our local window approach and ASpanformer

**Figure 10:**
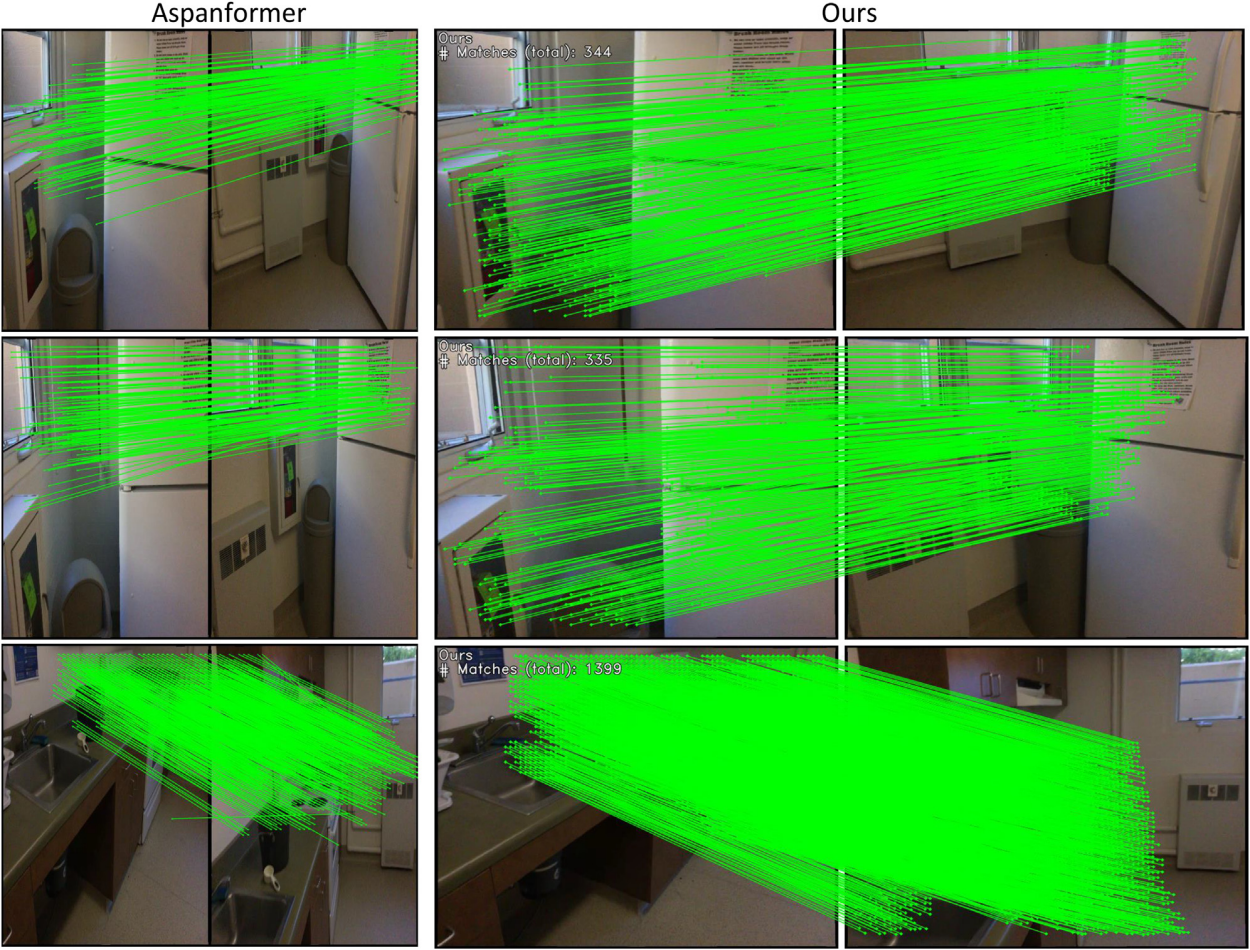
Comparison of feature matching effects between our method and the Aspanformer method

Our method differs from the local attention mechanism in ASpanformer^16^ in several ways. Firstly, ASpanformer’s^16^ local attention is applied in cross-attention, while our local attention is used in self-attention. We believe that in feature matching, local attention should not be used in cross-attention because cross-attention compares the features of two images for matching. If local attention is applied at this stage, it will prevent the two images from perceiving each other’s global context. For instance, region A in image 1 corresponds to region B in image 2, but we do not know which regions correspond before performing cross-attention. Therefore, local cross-attention might end up processing region A in image 1 with region D in image 2, resulting in poor matching outcomes. We compared the feature matching results of ASpanformer^16^ and our method using data from ScanNet. As shown in Figure 10, the feature points matched by our method are evenly distributed, while those matched by ASpanformer^16^ are biased toward the upper half of the image. Additionally, our method matches more feature points. (The image sizes for the results of the two methods differ because ASpanformer’s^16^ code resizes the images to 352 × 512 while we resize them to 640 × 480). Secondly, the position where we use local attention is different. We use it during the feature extraction stage, whereas ASpanformer^16^ uses it during the feature matching stage after the extracted features. Thirdly, our method has a fixed window size, while ASpanformer’s^16^ window size varies based on the uncertainty of the corresponding coordinates.

### Conclusion

This study proposes a feature-matching method based on local window aggregation. Considering that a global Transformer may lead to inconsistent matching results, we designed a module for local window aggregation, which combines local windows with global attention intersection. This approach ensures local consistency without being influenced by irrelevant regions to enable the detection of more correspondences. We designed a matching module progressing from coarse to fine to obtain more accurate matching results. Coarse matches are obtained through cross-attention applied to coarse-grained features, which are fused with fine-grained features using cross-attention. Refinement is performed on the fine-grained level using the coarse matching results to achieve the ultimate sub-pixel level matching accuracy. Under the same training conditions, our method improves AUC@20° in indoor pose estimation by 1.14%, precision in outdoor pose estimation by 1.8%, AUC@10px in homography estimation by 2.5%, and accuracy on DUC2 in visual localization by 1.65%, achieving the best results. In matching weak texture areas, our method has the highest number of matches, with an increase of 23.53%. Under lighting conditions, our method demonstrated the best matching accuracy from 1 pixel to 10 pixels. Our method exhibited higher matching accuracy in viewpoint changes than similar end-to-end methods. Considering different lighting and viewpoint changes below a 5-pixel threshold, our method displayed the highest matching accuracy, with higher precision in the 6 to 10-pixel range compared to similar end-to-end methods.

### Limitations of the study

**Figure 11:**
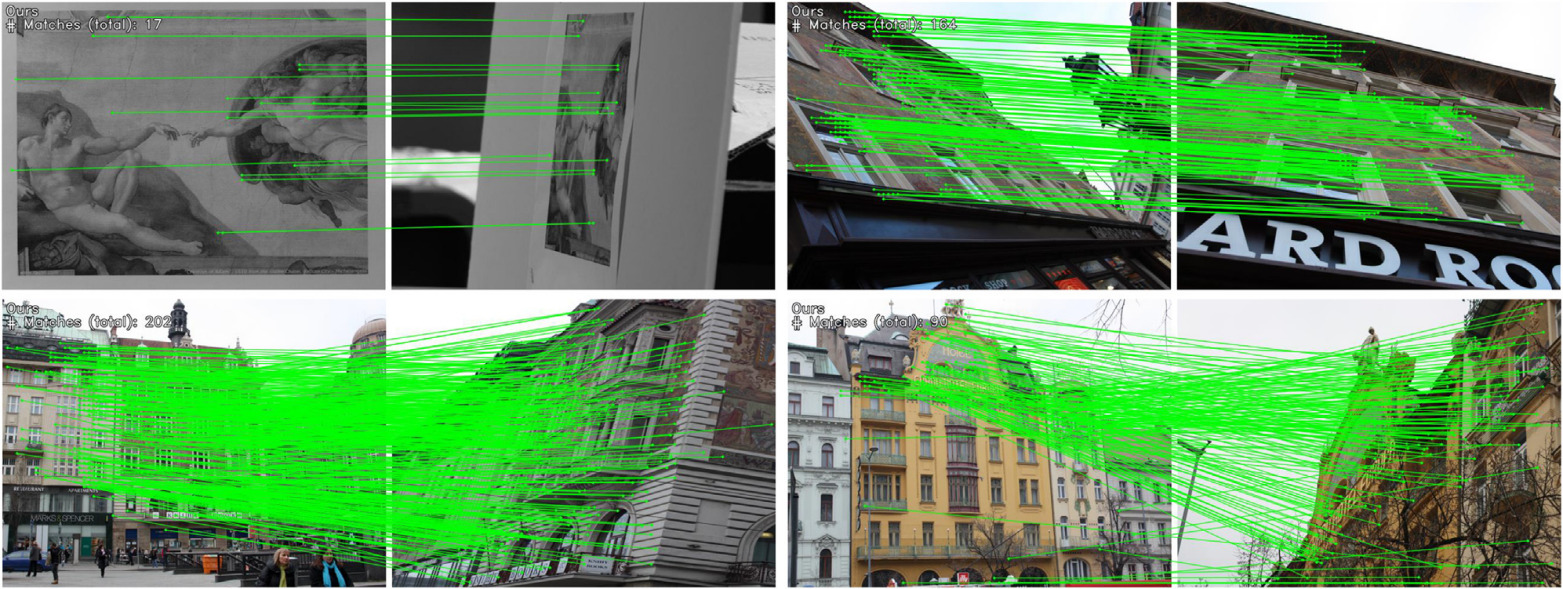
Feature matching under extreme viewpoint changes

Our method has limited capability in training high-resolution images. Therefore, our future work will focus on reducing the computational complexity of training high-resolution images without compromising matching performance. A common issue with machine learning-based methods is poor feature matching performance under extreme viewpoint changes. Our method also exhibits suboptimal feature matching under extreme viewpoint changes, as shown in Figure 11. Although our method can perform matching, the number of matched feature points is relatively low. This may be because our method did not use a dataset with exaggerated viewpoint changes during training, which hindered our model from extracting handy features and resulted in poor performance during the matching phase. Thus, our future research will also concentrate on feature matching under extreme viewpoint changes.

## Resource availability

### Lead contact

Further information and requests for resources and reagents should be directed to and will be fulfilled by the lead contact, Wenpeng Li (leewenpeng@126.com).

### Materials availability

This study did not generate new unique reagents.

### Data and code availability

• Data of the main experiment: https://osf.io/ds5r9/.
• TATA code to reproduce the statistical analyses and figures: https://osf.io/ds5r9/.
• Any additional information required to reanalyze the data reported in this paper is available from the lead contact upon request.

## Acknowledgments

This work was supported partly by 10.13039/501100001809National Natural Science Foundation of China (61872204), Heilongjiang Province Natural Science Foundation (LH2021F056), Heilongjiang Provincial Education Department, grant/award(135509113), and Graduate Innovation Research Project of Qiqihar University(authorization number QUZLTS_CX2023007).

## Author contributions

Conceptualization, Y.G., W.L., P.Z., and L.W.; methodology, Y.G., W.L., P.Z., and L.W.; investigation Y.G., W.l., and L.W.; data curation, Y.G. and W.l.; visualization, P.Z. and L.W.; formal analysis, Y.G. and W.L.; writing—original draft, Y.G. and W.L.; writing—review and editing, Y.G. and W.L.; funding acquisition Y.G.; supervision, W.L., P.Z., and L.W.

## Declaration of interests

The authors declare no competing interests.

## STAR★Methods

### Key resources table

**Table:** 

REAGENT or RESOURCE	SOURCE	IDENTIFIER
**Deposited data**		

Data	This paper	https://osf.io/ds5r9/
Original Code	This paper	https://osf.io/ds5r9/

**Software and algorithms**		

Python (version 3.8)	Python Software Foundation	https://www.python.org/
Numpy(version1.24.3)	Python package	https://numpy.org
PyTorch (version 1.13.1)	Python package	https://pytorch.org/
DGL Cuda11.6(version 0.9.1)	Python package	https://docs.dgl.ai/en/latest/install/
opencv-python (4.4.0.46)	Python package	https://github.com/skvark/opencv-python

### Experimental model and study participant details

The core objective of feature matching is establishing correspondences between two images. While current feature-matching methods without detectors have achieved impressive results, they often prioritize global features, neglecting regions with subtle texture variations, resulting in fewer matching points, especially in regions with weak textures. This paper proposes a feature-matching method based on local windows aggregation, which considers global features and focuses more on texture variations within local windows to achieve more accurate matching points and more accurate correspondences in regions with weak textures. Our method first employs a local window aggregation module to reduce irrelevant interference by applying window attention processing, followed by global attention processing, and generates coarse-grained and fine-grained feature maps. Subsequently, these feature maps are further processed by a matching module, and coarse-grained matches are obtained using the nearest neighbor principle. Finally, the coarse-grained matching results are refined on fine-grained feature maps after fusing coarse-grained and fine-grained feature maps, using local window refinement to obtain the ultimate matching results. Experimental results demonstrate that our method outperforms state-of-the-art methods in pose estimation, homography estimation, and visual localization under the same training conditions.

### Method details

#### Processing procedure

Our method mainly consists of three modules: the local windows aggregation module, the Coarse Matching module, and the Fine Matching module. Below, we briefly introduce the entire process. Given images $I^{A}$ and $I^{B}$, first, we extract multi-scale feature maps for each image using the local windows aggregation module. We denote the feature map of size 1/i as $F^{1/i}=\left\{F_{A}^{1/i},F_{B}^{1/i}\right\}$. Next, we input $F^{1/8}$ into the Coarse Matching module for coarse-grained feature matching. We use the nearest neighbor principle to obtain a confidence matrix $P_{c}$ and predict coarse-grained matches $M_{c}$ based on a confidence threshold. Finally, we input $F^{1/2}$, $F^{1/8}$ and the coarse-grained matches $M_{c}$ into the Fine Matching module. We upsample the $F^{1/8}$ and fuse them with the $F^{1/2}$ before performing fine-grained matching. We crop local windows from the $F^{1/2}$ and compute the spatial expectation coordinates of the two-dimensional heatmap for each local window to obtain the final matching results $M_{f}$.

The local windows aggregation (LWA) module takes the input image $I\in R^{2\times H\times W\times 1}$ (2, H, W, 1, representing the number of images, height, width, and the number of channels) and undergoes four rounds of local window aggregation processing and feature pyramid network (FPN) processing to obtain $F^{1/2}$ and $F^{1/8}$. The $C_{0}$, $C_{1}$, $C_{2}$, and $C_{3}$ represent the dimensions of the feature maps. Initially, the image is a grayscale image with only one dimension. After the first local window aggregation, the feature dimension changes from 1 to $C_{0}$, halving the height and width. After the second local window aggregation, the feature dimension changes from $C_{0}$ to $C_{1}$, and the height and width are halved again. After the third local window aggregation, the feature dimension changes from $C_{1}$ to $C_{2}$, and the height and width are halved again. After the fourth local window aggregation, the feature dimension changes from $C_{2}$ to $C_{3}$, and the height and width are halved once more. Finally, the four feature maps are fused through a feature pyramid, outputting two feature maps for subsequent coarse-grained and fine-grained feature matching. The dimensions of these two feature maps are $C_{0}$ and $C_{2}$, respectively. We denote the processing at each stage as $LWA_{i}\left(\cdot \right)$ and the FPN as FPN(·). The local window aggregation processing is represented as:

(Equation 11) $$I_{i}=LWA_{i}\left(I_{i}\right),i=1,2,3,4$$

(Equation 12) $$\left\{\left(F_{A}^{1/8},F_{B}^{1/8}\right),\left(F_{A}^{1/2},F_{B}^{1/2}\right)\right\}=FPN\left(I_{i}\right),i=1,2,3,4$$

Finally, we obtain feature maps of original image sizes 1/8 and 1/2.

In local window aggregation, the attention mechanism plays a central role. Ordinary attention calculation requires three inputs: Q (query), K (key), and V (value). The attention output is a weighted sum, where the weight matrix is determined by Q and its corresponding K. This process can be described as:

(Equation 13) $$Attention\left(Q,K,V\right)=SoftMax\left(QK^{T}\right)V$$

However, in visual tasks, the size of the weight matrix $SoftMax\left(QK^{T}\right)V$ grows quadratically with the increase in image resolution. When the image resolution is high, ordinary attention’s memory and computational costs become prohibitive. To address this issue, linear attention has been proposed,^22^ which replaces the softmax operation with the product of two kernel functions:

(Equation 14) $$Linear\_attention\left(Q,K,V\right)=\phi \left(Q\right)\left(\phi \left(K^{T}\right)V\right)$$

Here, ϕ(·)=elu(·)+1. Since the number of feature channels is much smaller than the number of pixels, the computational complexity decreases from quadratic to linear. Therefore, we adopt linear attention for global attention.

The local attention mechanism is inspired by the Swin Transformer,^40^ but unlike it, we do not use the shifting window operation and instead replace it with global attention. Additionally, we do not require the image height and width to be exact multiples of the window size; if they are not divisible, we pad the image accordingly. For an image input of size H×W×C, we first reshape it into a feature map of size $\frac{HW}{M^{2}}\times M^{2}\times C$ and divide this feature map into M×M non-overlapping local windows, where $\frac{HW}{M^{2}}$ represents the number of windows. Then, self-attention is computed separately for each window. The matrix calculation method for local window features $X\in R^{M^{2}\times C}$ is specific and used to implement local attention:

(Equation 15) $$Q=XP_{Q},K=XP_{K},V=XP_{V}$$

where $P_{Q},P_{K},P_{V}$ are projection matrices shared across different windows. Typically, $Q,K,V\in R^{M^{2}\times d}$. The attention matrix calculated through the self-attention mechanism within the local window is:

(Equation 16) $$Attention\left(Q,K,V\right)=SoftMax\left(QK^{T}/\sqrt{d}+B\right)V$$

where d represents the number of heads in multi-head attention. We denote this as W-MSA, which stands for window multi-head attention mechanism.

#### Matching steps

The steps for using the model to perform matching are as follows. First, read the two images to be matched, Image *A* and Image *B*, and convert the data of the two images into tensor types $tensor_{A}$ and $tensor_{B}$. Second, construct a dictionary object named data and place $tensor_{A}$ and $tensor_{B}$ into the dictionary with “image0” and “image1” as the keys. Third, pass the data object into the model for prediction. Fourth, the final prediction results are stored in the data object, where “mkpts0_f” and “mkpts1_f” are the coordinates of the feature matches for Image *A* and Image *B*, respectively, and “mconf” is the confidence level of the feature matches.

### Quantification and statistical analysis

All statistical details and sample sizes are provided. The exact statistical tests and variables used are described in the text and the legends of the tables and figures.
